# Unearthing the secrets of ancient immature insects

**DOI:** 10.7554/eLife.03443

**Published:** 2014-06-24

**Authors:** Enrique Peñalver, Ricardo Pérez-de la Fuente

**Affiliations:** 1 **Enrique Peñalver** is at the Museo Geominero, Instituto Geológico y Minero de España, Madrid, Spaine.penalver@igme.es; 2 **Ricardo Pérez-de la Fuente** is at the Museum of Comparative Zoology, Harvard University, Cambridge, United Statesperezdelafuente@fas.harvard.edu

**Keywords:** fossil, Diptera, Jurassic, China, none

## Abstract

Jurassic fossils of a bizarre fly larva that lived in water as a blood-sucking parasite highlight how much can be learnt from the study of the fossils of immature insects.

**Related research article** Chen J, Wang B, Engel MS, Wappler T, Jarzembowski EA, Zhang H, Wang X, Zheng X, Rust J. 2014. Extreme adaptations for aquatic ectoparasitism in a Jurassic fly larva. *eLife* **3**:e02844. doi: 10.7554/eLife.02844

**Image** Anterior part of a fossil insect larva discovered by Chen et al.

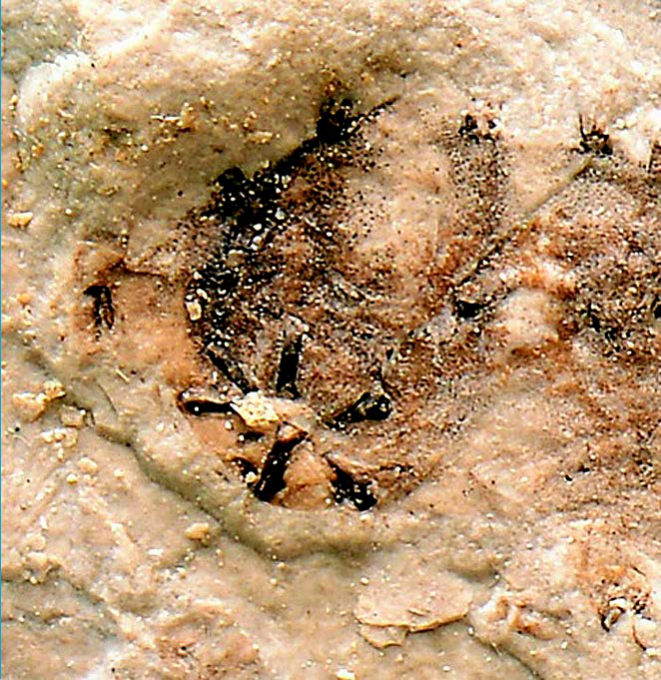

Insects are a highly diverse group of organisms, ranging from tiny fleas to creatures as large as some butterflies and moths. However, these very different insects have much in common. In particular, in around 80% of insect species, the egg hatches to become a soft-bodied larva that does not have wings, which then becomes a pupa, which finally becomes an adult insect. It has been suggested that the emergence of the larval stage was a hugely important innovation in the evolution of insects because, for example, larvae are able to exploit resources that are not used by adult insects (Grimaldi and Engel, 2005). However, the degree to which this so-called 'complete' form of metamorphosis explains the success of these insects is still unknown.

Insect larvae are very rarely found in the fossil record as body remains, partly because they contain relatively few hardened structures. Instead, traces of their activity fossilize more often, such as feeding marks on leaves or larval cases. Moreover, when found, body fossils of insect larvae are also challenging to study because insects tend to exhibit fewer traits during the larval and pupal stages of their life cycle than they do when they are adults.

But can the fossils of insect larvae provide us with novel insights into insect evolution and palaeobiology? Now, in *eLife*, Jun Chen, Bo Wang, Michael Engel and co-workers demonstrate that the answer to this question is “yes” by reporting the discovery of five exquisitely preserved, virtually complete, larval specimens all belonging to the same fly species (Chen et al., 2014). The specimens were found in Chinese fossil deposits that date back to the Middle Jurassic Epoch. Chen et al. were able to completely reconstruct the morphology of the larvae to reveal how they were uniquely, and somewhat bizarrely, adapted to life as aquatic ectoparasites. The name chosen for the new species—*Qiya jurassica*—reflects its unusual characteristics ('qiya' means bizarre in Chinese).

So what was *Qiya jurassica* like? Imagine a worm-shaped creature that has a tiny head equipped with heavily hardened mandibles, a thorax that bears a large ventral sucker armed with radially arranged 'teeth', short legs with bunches of spines on the back, and tentacle-like extensions on its rear end… Such a larval morphology is bizarre, even for insects!

Chen et al. demonstrate that *Qiya jurassica* larvae were aquatic and are related to water snipe flies (family Athericidae). This small group of flies, with about 100 known species, is related to horse flies, and the adults of both groups are infamous for their painful bites as they feed on blood (a habit that is known as hematophagy). However, unlike modern athericid larvae, the fossil larvae exhibit a combination of traits adapted to hematophagy and ectoparasitism (which involves an organism spending a significant part of its life cycle on its host; Balashov, 2006). These adaptions include the thoracic sucker and legs with spines for anchoring to its host, and piercing-sucking mandibles for fluid feeding.

It is clear that feeding on blood as an ectoparasite evolved several times in insects (Figure 1). Ectoparasites not only feed on blood but also on other animal substances, such as gland secretions, and keratin from feathers, hair or skin. Although insects that are both ectoparasites and blood-feeders are very unusual in the fossil record, 'free-living' insects that feed on blood, such as mosquitoes, are relatively abundant (Lukashevich and Mostovski, 2003). The most striking example of the latter is the fossil of a 46-million-year-old female mosquito in which its blood meal is preserved (Greenwalt et al., 2013).

**Figure 1.**
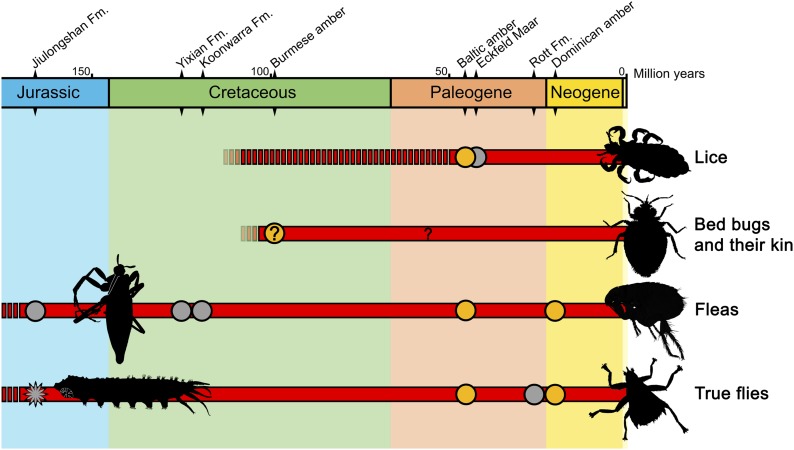
A timeline of the insect lineages that are ectoparasitic and feed on blood. The groups to which they belong are shown on the right. Temporal ranges (shown in red) are based on the fossil record: grey dots represent fossils found in compression (rock) deposits, orange dots those found in amber; temporal ranges not supported by fossil evidence are denoted by a broken red line. The new fossil fly larva reported by Chen et al. is shown as a star-shaped grey dot. Insect outlines to the right depict living forms, those on the left extinct forms. The question mark denotes a fossil with unclear affiliations and life habits. Quaternary records (for the last 2.5 million years) are not shown. Sources: Lukashevich and Mostovski, 2003; Grimaldi and Engel, 2005; Grimaldi and Engel, 2006; Engel, 2008; Huang et al., 2012.

By taking into account other fossils found in the same outcrop where the *Qiya jurassica* specimens were collected, Chen and co-workers—who are based at Linyi University, the University of Bonn, the Chinese Academy of Science, the University of Kansas and the Natural History Museum—conclude that a likely host of *Q. jurassica* larvae were aquatic salamanders.

Although no blood-sucking ectoparasites are known for extant reptiles or amphibians (Grimaldi and Engel, 2005), it was recently discovered—also from Chinese deposits—that Mesozoic reptiles were most likely ectoparasitized by giant fleas (Huang et al., 2012; Figure 1). Thus, the findings of Chen et al. lead to the novel idea that amphibians could have been ectoparasitized by blood-sucking insects in the past.

The evolution of larval stages is one of the most overlooked topics in insect palaeontology. The research of Chen et al. highlights what can be discovered from studying larval fossils. As insect palaeontology, despite its remarkable progress in the past decades, remains strongly biased towards the study of adult forms, there is a clear need to train new researchers to recognize and study the fossils of immature insect stages. Such an undertaking will help us to understand the role played by the larval and pupal stages through evolutionary time, and learn more about the most ecologically dominant animal lineage on land.
